# Perseveration in a spatial-discrimination serial reversal learning task is differentially affected by MAO-A and MAO-B inhibition and associated with reduced anxiety and peripheral serotonin levels

**DOI:** 10.1007/s00213-017-4569-x

**Published:** 2017-03-02

**Authors:** Peter Zhukovsky, Johan Alsiö, Bianca Jupp, Jing Xia, Chiara Guiliano, Lucy Jenner, Jessica Griffiths, Errin Riley, Sajeed Ali, Angela C. Roberts, Trevor W. Robbins, Jeffrey W. Dalley

**Affiliations:** 1 Behavioural and Clinical Neuroscience Institute and Department of Psychology, University of Cambridge, Cambridge, UK; 2 Department of Neuroscience, University of Uppsala, Uppsala, Sweden; 3 Florey Institute of Neuroscience and Mental Health, University of Melbourne, Parkville, VIC Australia; 4 Department of Physiology, Development and Neuroscience, University of Cambridge, Cambridge, UK; 5 Department of Psychiatry, University of Cambridge, Cambridge, UK; 6 Department of Psychology, University of Cambridge, Downing Street, Cambridge, CB2 3EB UK

**Keywords:** Behavioral flexibility, Moclobemide, Lazabemide, Endophenotype, Orbitofrontal cortex, Basolateral amygdala, Striatum

## Abstract

**Rationale:**

Impairments in behavioral flexibility lie at the core of anxiety and obsessive-compulsive disorders. Few studies, however, have investigated the neural substrates of natural variation in behavioral flexibility and whether inflexible behavior is linked to anxiety and peripheral markers of stress and monoamine function.

**Objective:**

The objective of the study was to investigate peripheral and central markers associated with perseverative behavior on a spatial-discrimination serial reversal learning task.

**Methods:**

Rats were trained on a reversal learning task prior to blood sampling, anxiety assessment, and the behavioral evaluation of selective monoamine oxidase-A (MAO-A) and MAO-B inhibitors, which block the degradation of serotonin (5-HT), dopamine (DA), and noradrenaline (NA).

**Results:**

Perseveration correlated positively with 5-HT levels in blood plasma and inversely with trait anxiety, as measured on the elevated plus maze. No significant relationships were found between perseveration and the stress hormone corticosterone or the 5-HT precursor tryptophan. Reversal learning was significantly improved by systemic administration of the MAO-A inhibitor moclobemide but not by the MAO-B inhibitor lazabemide. Moclobemide also increased latencies to initiate a new trial following an incorrect response suggesting a possible role in modulating behavioral inhibition to negative feedback. MAO-A but not MAO-B inhibition resulted in pronounced increases in 5-HT and NA content in the orbitofrontal cortex and dorsal raphé nuclei and increased 5-HT and DA content in the basolateral amygdala and dorsomedial striatum.

**Conclusions:**

These findings indicate that central and peripheral monoaminergic mechanisms underlie inter-individual variation in behavioral flexibility, which overlaps with trait anxiety and depends on functional MAO-A activity.

## Introduction

Behavioral inflexibly is common to a range of compulsive and anxiety-related brain disorders, including addiction, obsessive-compulsive disorder (OCD), and schizophrenia (Fineberg et al. 2010; Robbins et al. 2012; Voon et al. 2015). Elucidating the neural and psychological mechanisms of behavioral inflexibility is therefore important to facilitate the diagnosis and treatment of a range of mental disorders. Based on selective brain intervention studies, much is known about the neural mechanisms underlying one aspect of impaired behavioral flexibility, namely excessive perseveration in response to shifts in the stimulus-reward contingency of reversal learning paradigms (Castane et al. 2010; Rygula et al. 2010). However, few studies have investigated the neural mechanisms of inter-individual differences in behavioral flexibility and how these relate to anxiety and other traits present in OCD and related disorders.

Convergent evidence indicates that serotonin (5-HT) modulates reversal learning in a number of species (Roberts 2006). As reviewed by Izquierdo et al. (2016), elevated post synaptic 5-HT activity facilitates reversal learning (Bari et al. 2010; Barlow et al. 2015; Danet et al. 2010; Wallace et al. 2014) whereas reduced 5-HT signaling impairs reversal learning and increases perseveration (Clarke et al. 2005, 2007; Lapiz-Bluhm et al. 2009; Rygula et al. 2015). In a similar manner, selective 5-HT_2A_ and 5-HT_2C_ receptor antagonists, respectively, impair and improve reversal learning (Boulougouris et al. 2008) with the orbitofrontal cortex (OFC) an important locus for the latter beneficial effects (Boulougouris and Robbins 2010), consistent with much previous evidence implicating the OFC in reversal learning (Boulougouris et al. 2007; Dias et al. 1996; Schoenbaum et al. 2009; Stalnaker et al. 2009). Functionally, 5-HT in this region is hypothesized to inhibit actions to previously rewarded stimuli when aversive or negative outcomes are expected (Cools et al. 2011; Roberts 2011).

We recently reported in outbred rats that excessive perseveration on an appetitive, spatial reversal learning task is associated with diminished 5-HT metabolism and 5-HT_2A_ receptor availability in the OFC, as well as altered gene expression of the two isoforms of monoamine oxidase, MAO-A and MAO-B, in the dorsal raphé nucleus (DRN) and OFC (Barlow et al. 2015). In the present study, we extended these findings by investigating the causal involvement of MAO-A and MAO-B in mediating reversal learning performance. We reasoned that since MAO-A has a high affinity for 5-HT and norepinephrine (NE), unlike MAO-B (Da Prada et al. 1988; Shih and Thompson 1999), selective MAO-A inhibition by moclobemide would improve behavioral flexibility. We extended our analysis to the measurement of 5-HT in blood samples since MAO inhibition produces parallel increases in 5-HT levels in the brain and blood (Malyszko et al. 1993), similar to the effects of psychostimulants and selective 5-HT reuptake inhibitors (Zolkowska et al. 2006). In addition, platelet MAO activity has been proposed as an index of central 5-HT activity (Stahl 1985) and low activity of this enzyme has been associated with OCD severity (Arrojo et al. 2007). Thus, the peripheral measurement of unbound 5-HT may be an accessible marker of central 5-HT transmission, under some circumstances, and reflect inter-individual differences in behavioral flexibility. We also measured levels of the 5-HT precursor tryptophan, as well as circulating levels of the stress hormone corticosterone. Subsequently, we used a factor analysis to relate these levels to trait anxiety and perseverative errors on a spatial reversal learning task (Barlow et al. 2015).

The primary objective of the present study was to clarify the extent to which individual variation in behavioral flexibility on a spatial-discrimination serial reversal learning task can be explained by peripheral biomarkers and trait anxiety and to relate these trait markers to levels of 5-HT and other monoamines in key brain loci implicated in reversal learning, including the OFC, basolateral amygdala (BLA), and striatum (Izquierdo et al. 2013; Ochoa et al. 2015).

## Methods

### Subjects

**Fig. 1:**
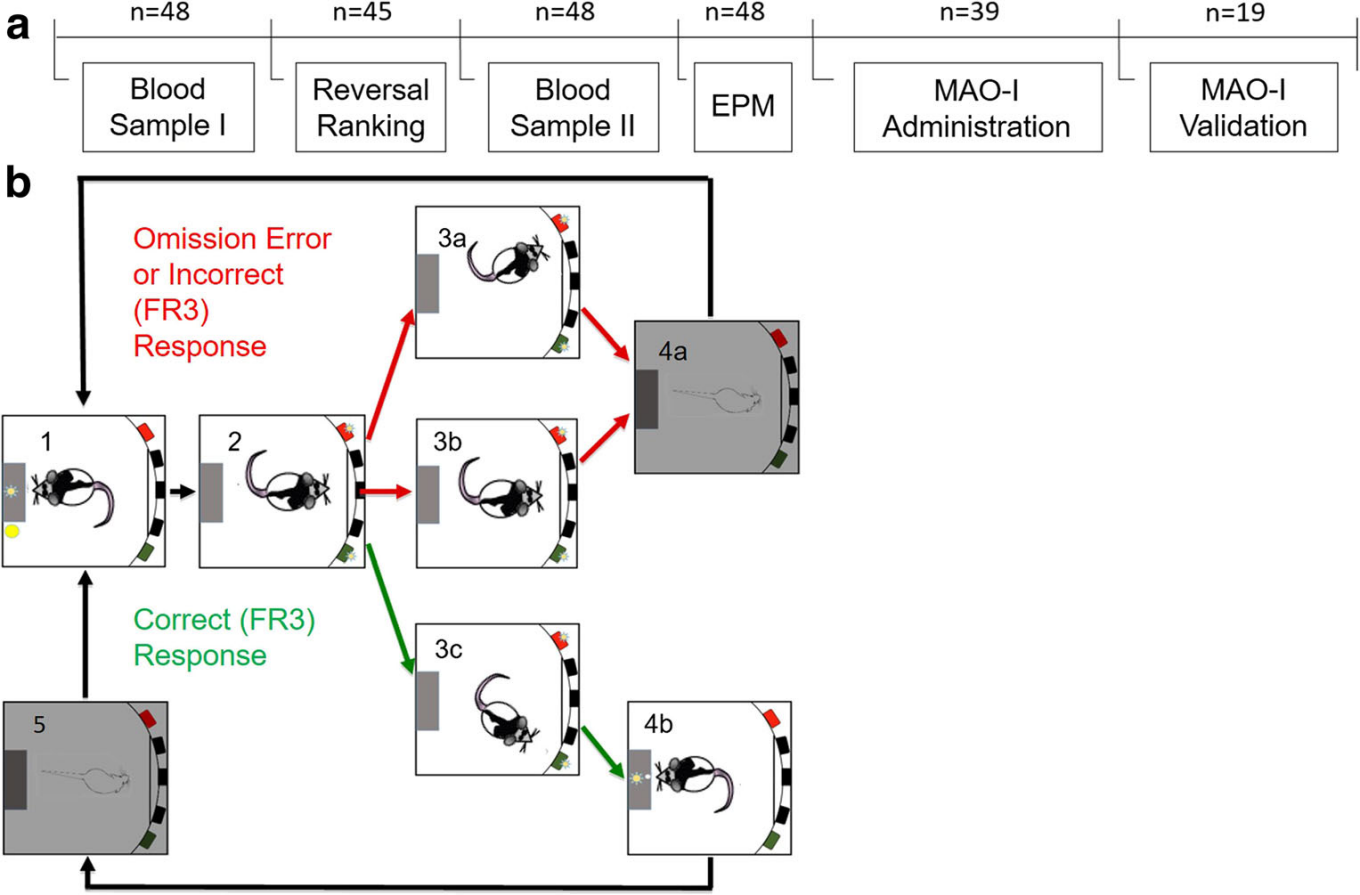
**a** Experimental timeline and group sizes. Blood samples were extracted before and after training on the reversal learning task followed by anxiety testing and the behavioral and neurochemical evaluation of MAO inhibition. **b** Schematic depiction of the spatial-discrimination reversal learning task. Rats initiated each session by making a nose-poke response in the food magazine (*1*). By making nose-poke responses in the “correct” aperture under a fixed-ratio-3 schedule of reinforcement, a food pellet was delivered in the illuminated magazine (*2*-*3c*; *4b*), followed by a 5-s time-out (*5*). “Incorrect” responses and failure to respond (“omissions”) resulted in a 5-s time-out (*4a*). If the rat achieved nine correct responses over the previous 10 trials, the reward contingencies were switched such that the rat now needed to respond at the previously unrewarded aperture. Each animal completed three reversals within a 1-h session

Male Lister-hooded rats (*n* = 48) weighing 290 ± 17 g at the beginning of experiments were used (Charles River, Kent, UK). They received 18 g of laboratory chow once a day with ad libitum access to water. The weight of each animal was recorded each week with animals maintained at 85–95% of free-feeding weights. When no behavioral training or testing took place, rats received 20 g of chow per day. All animals were housed in groups of four per cage and kept under a reversed 12 h light/dark cycle (lights off 07:00 h until 19:00 h). Rats were trained on the spatial reversal learning task between 14:00 and 19:00 h. Testing on the elevated plus maze (EPM) and the collection of blood samples took place between 15:30 and 16:30 h. Five rats were excluded from the study because they failed to reach criterion on the reversal learning task. One further animal developed audiogenic seizures and therefore was culled before drug challenge. Forty-two animals received systemic drug injections, of which 19 animals were used for region-specific post mortem monoamine analysis to validate the effects of moclobemide and lazabemide (Fig. 1a). Three other animals failed to complete the task after drug administration and therefore were excluded from further analysis. Experiments complied with the UK Animals (Scientific Procedures) Act of 1986 and were approved by the ethics review committee at Cambridge University.

### Behavioral apparatus

Twelve five-hole operant chambers (Med Associates, Georgia, VT) controlled by two computers and Whisker Control software (Cardinal and Aitken 2010) were used (Fig. 1b). Each chamber was enclosed in a ventilated sound-attenuating box, fitted with five apertures in a curved wall and a food magazine on the opposite wall of the box that delivered rodent sugar pellets (TestDiet®, Purina, UK). A yellow light-emitting diode stimulus was placed at the rear of each aperture. The middle three apertures were blocked using a metal plate and were not part of the experimental setup. The food magazine and entire chamber was illuminated by light emitting diodes. Infrared beams detected responses in the magazine and apertures.

The elevated plus maze was constructed from black Perspex and consisted of a central platform surrounded by two open arms and two enclosed arms in the shape of a plus sign (Molander et al. 2011; Walf and Frye 2007). The plus-shaped platform was elevated 60 cm above the floor in a room illuminated by white light (intensity 70 lx). Exploratory behavior in the maze was recorded and monitored on a ceiling-mounted Yi Action Camera connected wirelessly to a computer**.**

### Behavioral training

Training began with 2 days of habituation during which animals were exposed to the testing boxes with all cue lights, magazine, and house lights on for 20 min. They were encouraged to explore the apparatus by baiting the response apertures and magazine with sugar pellets. Before the start of each session, all lights were extinguished. The first trial was initiated by the animal making a nose-poke in the magazine, which triggered the illumination of a cue light in each aperture. Responding in either aperture was initially reinforced by the delivery of a single food pellet. Task difficulty was then progressively increased with just one aperture reinforced (the “correct” aperture) under FR1, FR2, and FR3 schedules (see Table 1). Finally, the inter-trial interval (ITI) was gradually increased from 1 to 2 s and finally to 5 s upon completion of the previous stage. For all training stages, a criterion of 50 correct trials was required to proceed to the next level. While the FR1-FR3 stages were restricted to 20 min, session times increased to 30 min for the ITI stages. If a subject made an incorrect response at the non-cued aperture, it was not penalized during training. However, a failure to make the appropriate response within 30 s of initiating a new trial was recorded as an omission and was followed by a 5-s time-out where all lights were turned off.

**Table 1:** Summary of the training procedure for the acquisition of the spatial-discrimination task and subsequent reversals of the stimulus-reward response contingency

Stage	Total time (min)	Time-out period	Criterion	Cues
Habituation	20	None	Eat all pellets in box	All lights on
FR1 pre-training	20	None	50 pellets	Both sides cued and rewarded
FR1, FR2, FR3 training	20	30 s	50 pellets	Only one side cued
1 s, 2 s, 5 s ITI training	30	None for 1 s ITI, otherwise 30 s	50 pellets	Only one side cued
Spatial-discrimination	60	30 s	9/10 correct on one side	Both sides cued
Reversal test	60	30 s	Three reversals, i.e., 9/10 correct on 4 sides	Both sides cued

*FR* fixed ratio, *ITI* inter-trial interval

In the spatial-discrimination task, the training setup above was modified with both apertures lit but with only one of apertures rewarded. Three nose-pokes in the “incorrect” aperture now resulted in the omission of reward and a 5 s time-out. Rats were given 1 h to complete the discrimination task by achieving 9 correct trials across previous 10 trials. If animals failed to achieve criterion after 2 days, they were retrained by completing the 5-s ITI condition within a single session.

On the day following the completion of the discrimination task, animals experienced the same configuration of the task, whereby the correct aperture was kept the same on both days as a measure of retention (Fig. 1b). Once the 9/10 criterion was achieved, the previously correct aperture was no longer rewarded and the rat was required to respond in the other aperture to obtain reward. Similar to the discrimination condition, an incorrect response or an omission resulted in a 5 s time-out. Subjects could complete up to three reversals during the 1-h session.

### Elevated plus maze

Animals were habituated to the experimental room for 30 min in their home cage before testing commenced. Each rat was placed on the central platform facing an open arm. The maze was thoroughly cleaned with water and dried between each test. Recordings during the first 6 min on the EPM were manually scored, specifically to record the time spent in the open arms and the number of entries made into the open arms, as described previously (Walf and Frye 2007).

### Systemic drug administration

Forty-two animals received mock injections 2 days before the start of the administration of the selective, reversible MAO-A and MAO-B inhibitors (moclobemide and lazabemide, respectively). Moclobemide and lazabemide hydrochloride were purchased from Tocris (UK) and dissolved in 15% hydroxypropyl-beta-cyclodextrin and 0.9% saline (“vehicle”). Moclobemide was fully dissolved using repeated sonication at +35 °C. Following the ranking of the animals by their reversal learning performance, two groups of animals were formed, matched for the number of perseverative errors made, and each assigned to one of the two MAO inhibitors. Given the relatively short washout periods for the drugs (Da Prada et al. 1988), each animal received four separate treatments across 3-day intervals, starting with a baseline retention session (day 1), a drug administration session (day 2), and a drug-free day. Doses for moclobemide (3 and 16 mg/kg, combination of 10 mg/kg of moclobemide and 10 mg/kg lazabemide) and lazabemide (1 and 10 mg/kg) were selected on the basis of previous literature (Da Prada et al. 1988; Jolkkonen et al. 2000; Kitaichi et al. 2006, 2010; Maki et al. 2000) and administered intraperitoneally (1 ml/kg). The dosing regimen followed a randomized modified Latin square design to control for training and crossover effects. One hour after the drug (or vehicle) injections, subjects were assessed for reversal learning performance.

In order to validate the effects of moclobemide and lazabemide on monoamine levels, 19 animals were matched for baseline performance and drug history and subsequently divided into three groups: a vehicle control group (15% HPB, *n* = 5), a lazabemide group (10 mg/kg, *n* = 6), and a moclobemide group (16 mg/kg, *n* = 4 and 3 mg/kg, *n* = 4) groups. Consistent with the timing of previous testing conditions, animals were culled for ex vivo neurochemical analysis 1 h after each injection.

### Blood analyses

Sublingual blood samples were collected in isoflurane-anesthetized animals (2.5% isoflurane in 95% O_2_, 5% CO_2_). Approximately 1 ml of blood was collected in tubes primed with ethylenediaminetetraacetic acid (EDTA), cooled on dry ice, and centrifuged at 3000×*g* for 20 min at 4 °C. Supernatant plasma was aliquoted in separate tubes for monoamine quantification using high-performance liquid chromatography (HPLC) with electrochemical detection (ECD). Plasma corticosterone was quantified by radioimmunoassay (Carter et al. 2004) using a citrate buffer at pH 3.0 to denature the corticosteroid-binding globulin and a specific corticosterone antibody (kindly supplied by G. Makara, Institute of Experimental Medicine, Budapest, Hungary), as previously described in detail (Atkinson et al. 2006; Windle et al. 1998).

### Monoamine analysis

Plasma samples were diluted 1:20 in 0.2 M perchloric acid, and centrifuged at 10,000 rpm at 4 °C for 20 min. Twenty-five microliters of the supernatant was injected onto the HPLC-ECD system to measure levels of 5-HT, 5-hydroxyindoleacetic acid (5-HIAA), tryptophan, and noradrenaline (NA), as described previously (Dalley et al. 2002). Detection and quantification were achieved using a Coulochem II detector with an analytical cell and two electrodes in series (E1 −250 mV, E2 +250 mV). The signal from E2 was integrated using computer software (Chromeleon, Dionex, UK).

One hour after drug injection, animals were killed by asphyxiation in a rising concentration of CO_2_ (*n* = 19). Brains were rapidly removed and flash frozen in liquid nitrogen, placed on dry ice, and stored at −80 °C. They were later cut into 150 μm coronal sections on a Jung CM300 cryostat (Leica, Wetzlar, Germany) and stored at −80 °C. At room temperature, small aliquots of tissue were removed bilaterally from two consecutive sections from the dorsomedial prefrontal cortex (dmPFC), OFC, DRN, hippocampal CA1 area, lateral hypothalamus (LH), BLA, dorsomedial striatum (dmS), and nucleus accumbens (NAcb) using a micropunch of diameter 1.0 mm (Fig. 6e). More details of this procedure can be found in Palkovits (1973). Samples were homogenized in 60 μl of 0.2 M perchloric acid using an ultrasonic cell disruptor, spun at 6000 rpm for 20 min (4 °C), and analyzed for 5-HT, NA, DA, 5-HIAA, and 3-4 dihydroxyphenylacetic acid (DOPAC). Monoamine levels were quantified in 25 μl of the homogenized brain samples using HPLC-ECD, as described above.

### Statistical analyses

Statistical analyses were conducted using SPSS for Windows (IBM version 23). Perseveration was assessed using the total number of trials and errors made until subjects achieved criterion. Errors were considered perseverative in nature if in a window of 10 trials, 7 incorrect responses were made. The number of perseverative errors made during the three reversals was used to rank the animals, consistent with (Barlow et al. 2015). Based on this ranking, three groups were formed that included highly perseverative (*n* = 11), mid-range (*n* = 20), and low-perseverative animals (*n* = 11). One of the low-perseverative rats and two of the mid-range rats were unable to complete the task in drug conditions and had to be excluded (Table 2). A mixed effects ANOVA was used to analyze within-subject effects of the drugs and between-subject effects of group as well as their interactions following systemic drug administration. Partial eta-squared (*η* ^2^) was used to assess effect size. A two-way between-subject ANOVA was used to compare the effects of the drugs on monoamine levels in the brain. If sphericity was violated (significant Mauchly’s test), a Greenhouse-Geisser correction was used. When significant main effects or interactions were found, post hoc analyses were carried out using Fisher’s LSD tests and the calculation of effect size *η* ^2^.

**Table 2:** Final group sizes for animals that successfully completed the task under drug conditions

	Moclobemide			Lazabemide		
Perseveration rank	High	Mid	Low	High	Mid	Low
Group size	5	9	4	6	9	6
Total size	18			21		

To identify markers of perseveration and anxiety, a factor analysis model was used. Since several variables were positively skewed and significantly non-normal, principal axis factoring was chosen as the integration method (Costello and Osborne 2005). Further, since the extracted factors did not correlate well with each other, the orthogonal rotation method (varimax) was preferred. Most errors made by the animals were perseverative; hence, only those were included to avoid excessive multicollinearity. Factor analysis variables included perseverative errors, total trials to criterion, and plasma levels of neurochemicals and corticosterone, alongside measures of trait anxiety (proportion of time spent in open arms of the EPM and percentage of open arm entries). Proportions were the preferred dependent variable to control for general locomotor activity (Walf and Frye 2007). Inferential contrasts were considered statistically significant at *α* = 0.05.

## Results

**Fig. 2:**
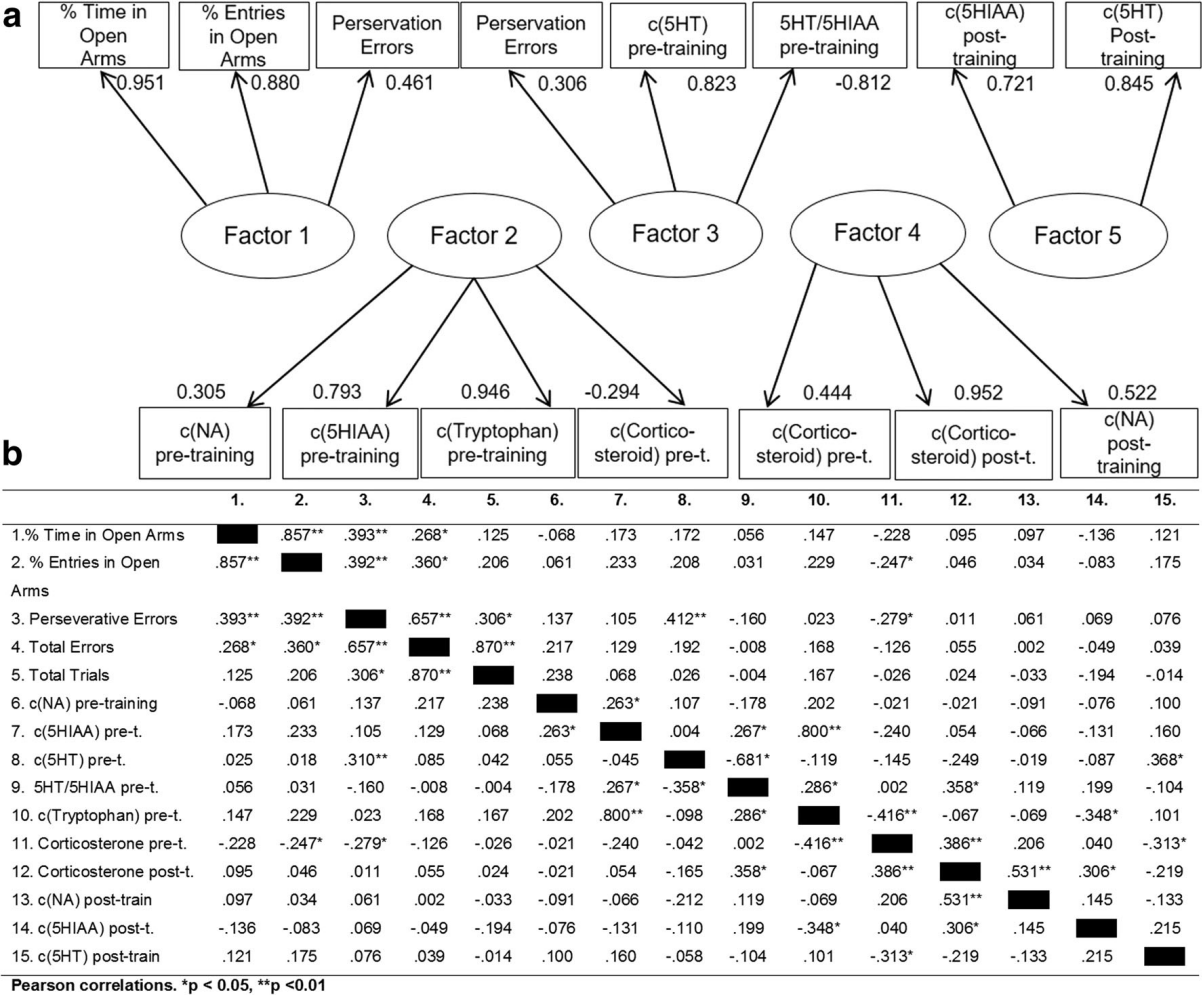
**a** Factor loadings (all loadings >0.3 are displayed). **b** Correlation matrix of all variables in the factor analysis, including peripheral monoamines, corticosterone, and behavioral measures

Means ± SEM perseverative errors were 36.3 ± 2.6 for high-perseverative animals, 19.6 ± 1.0 for the mid-range group and 7.3 ± 0.79 for the low-perseverative group. Within these high-, mid- and low-perseverative groups, the total number of errors (mean ± SEM: 58.9 ± 4.3, 37.6 ± 2.1, 31.5 ± 2.2, respectively) and total trials to criterion (mean ± SEM: 137.3 ± 10.3, 104.9 ± 6.0, 101.5 ± 6.5, respectively) followed the distribution of perseverative errors (Fig. 2). Prior to training, perseverative errors, total errors, and total trials to criterion as well as concentrations of peripheral NA, 5-HIAA, 5-HT, and tryptophan were positively skewed (skewness 0.82, 1.01, 0.97, 3.627, 1.0, 1.72, and 2.048, respectively). 5-HT and NE distributions remained skewed after training (1.70 and 0.73, respectively), similar to corticosterone levels and the 5-HT/5-HIAA ratio (1.71 and 1.52). Other variables were less skewed, as indicated by values below 0.7.

### Increased perseveration is associated with decreased anxiety and plasma 5-HT levels

**Fig. 3:**
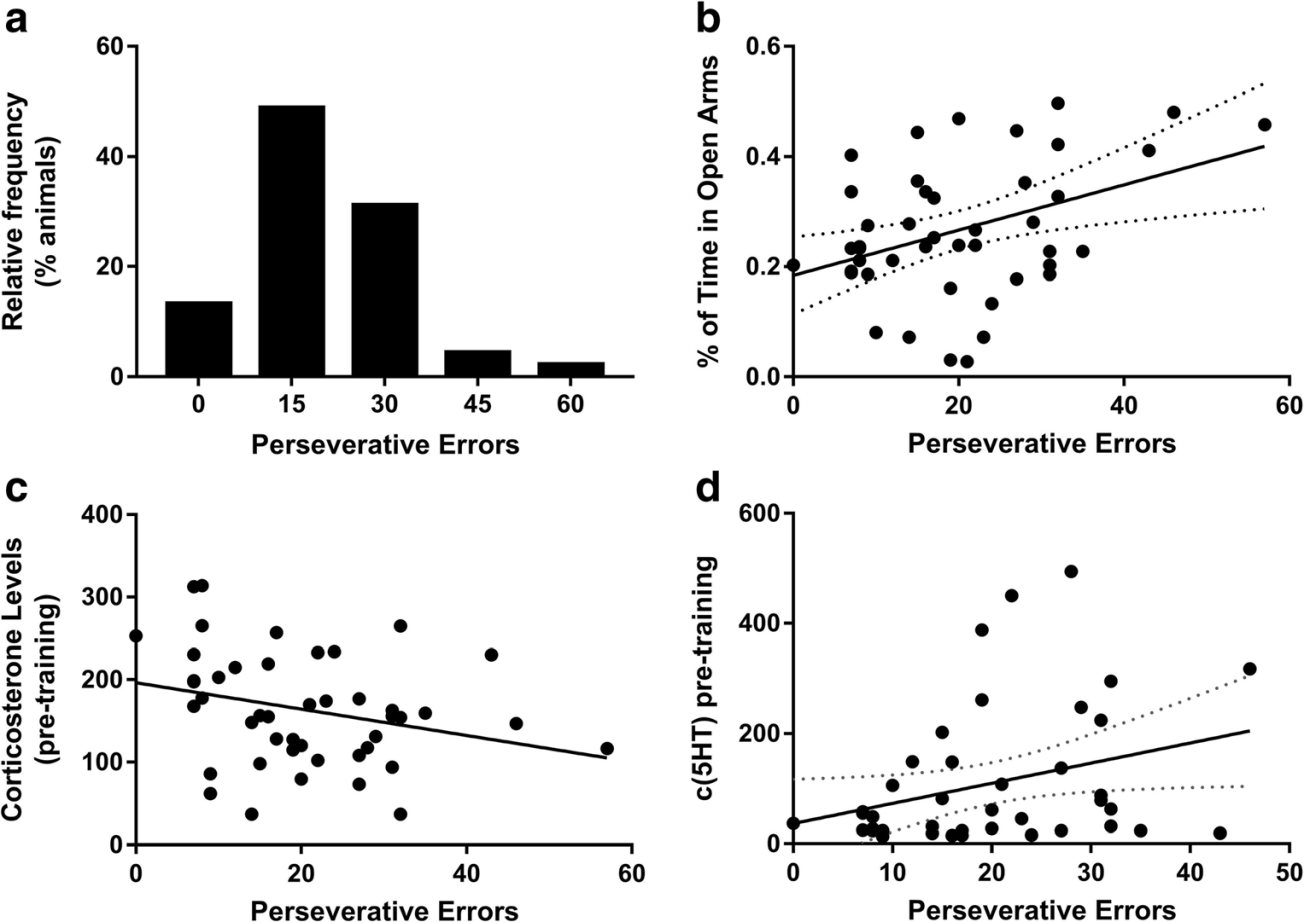
**a** Distribution of perseverative errors on the spatial-discrimination reversal learning task expressed as percentage of the cohort size (*n* = 45). **b** Perseverative errors were significantly correlated with the proportion of time spent in the open arms of the elevated plus maze (*r* ^2^ = 0.154, *p* = 0.008). **c** Lack of a significant relationship between perseverative errors and blood corticosterone levels (*r* ^2^ = 0.096, *p* = 0.064). **d** Positive relationship between the plasma concentration of 5-HT (in fmoles/μl) and the perseverative errors (*r* ^2^ = 0.096, *p* = 0.045)

Factor analysis was used to investigate the correlative relationships between plasma monoamine levels and task performance. Following the Kaiser criterion, all five factors with eigenvalues greater than one were extracted and orthogonally rotated that jointly accounted for over 58.2% of the variance in the data (Fig. 3). The first factor featured the two anxiety measures as well as perseverative errors. The second factor included pre-training levels of NA, 5-HIAA, tryptophan, and corticosterone. The third factor, accounting for 11.9% of variance, included perseveration, plasma levels of 5-HT, and the 5-HT/5-HIAA ratio. Corticosterone levels and post training levels of NA were loaded on factor 4 whereas post training levels of 5-HIAA and 5-HT were loaded on factor 5. Factors 4 and 5 jointly explained 8% of the variance.

Linear regression models were then created to investigate the strength of associations between perseveration, anxiety measures, and peripheral biomarkers. As shown in Fig. 3, a positive relationship was found between the proportion of entries into the open arms of the EPM and the perseverative errors (*F*_1,43_ = 7.82 *r* = 0.39, *p* = 0.008). Reflecting the loadings on the third factor, a significant correlation was found between perseverative errors and pre-training levels of 5-HT in the plasma (*F*_1,43_ = 4.27, *r* = 0.31, *p* = 0.045).

### MAO-A inhibition but not MAO-B inhibition improves reversal learning

**Fig. 4:**
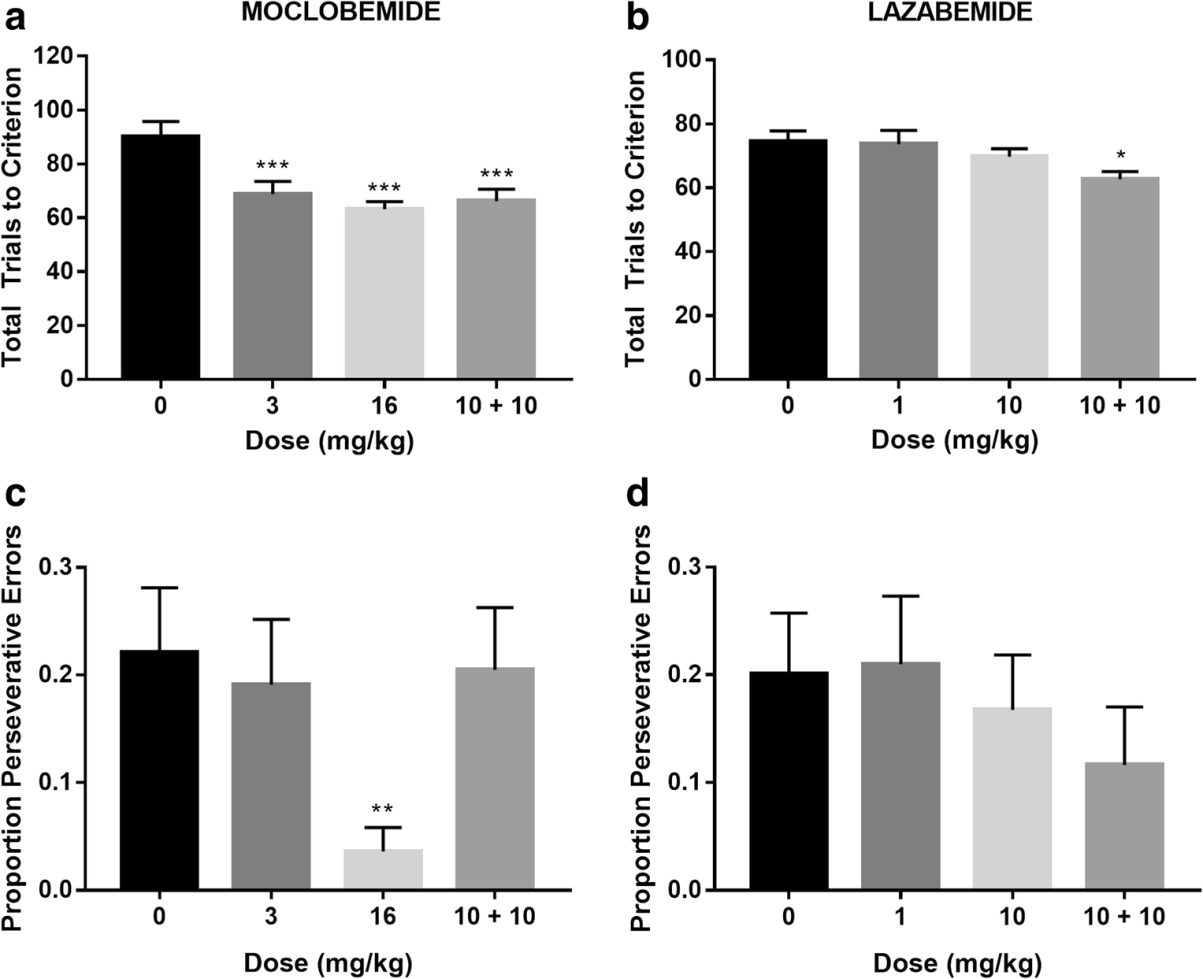
Effects of moclobemide (*n* = 18) and lazabemide (*n* = 21) on total trials to achieve criterion (**a**, **b**) and the proportion of perseverative errors (**c**, **d**). Mean values ± SEM for a single post drug administration session are shown. Significance is denoted as follows: *p < 0.05, **p < 0.01, ***p < 0.001 versus vehicle

The effects of MAO-A and MAO-B inhibition on reversal learning performance are shown in Fig. 4. As no interactive effects were found between the effects of moclobemide and perseveration group on total trials, errors, or proportion of perseverative errors (*F*_6,45_ = 1.32, *p* = 0.27; *F*_6,45_ = 0.40, *p* = 0.88, *F*_6,45_ = 0.84, *p* = 0.55, respectively), these were collapsed across perseveration group for subsequent analyses. This analysis revealed that moclobemide significantly improved reversal learning performance, as indexed by total trials to criterion (*F*_3,45_ = 11.27, *p* < 0.0001, *η* ^2^ = 0.429; Fig. 4a). Both the high and the low doses of moclobemide, as well as the combination of moclobemide and lazabemide, produced significant improvements compared with the vehicle group, as revealed by post hoc comparisons (*p* < 0.001, *η* ^2^ = 0.606; *p* < 0.0001, *η* ^2^ = 0.677; *p* < 0.002, *η* ^2^ = 0.486, respectively).

With respect to the lazabemide group, only the combined dose decreased the number of trials to achieve criterion (*F*_3,60_ = 3.33, *p* < 0.025, *η* ^2^ = 0.143; Fig. 4b). No significant interactive effects of lazabemide and perseveration group were observed on this measure. The combination of moclobemide and lazabemide decreased the number of trials to criterion compared with the high (*p* = 0.046, *η* ^2^ = 0.184) and low doses (*p* = 0.033, *η* ^2^ = 0.209) of lazabemide and the vehicle group (*p* = 0.023, *η* ^2^ = 0.234). Lazabemide itself had no significant effects on total trials to criterion.

Analysis of total errors mirrored the effects of moclobemide and lazabemide on total trials to criterion. Thus, one-way repeated measures ANOVA revealed significant main effects of drug treatment (moclobemide *F*_3,45_ = 7.51, *p* = 0.0001, *η* ^2^ = 0.344; lazabemide *F*_3,54_ = 4.83, *p* = 0.005, *η* ^2^ = 0.212); *post hoc* analyses identified significant effects of high and low doses of moclobemide, as well as the drug combination, to decrease the total number of errors compared with the vehicle group (*p* = 0.001, *η* ^2^ = 0.542; *p* = 0.001, *η* ^2^ = 0.535; *p* = 0.01, *η* ^2^ = 0.367, respectively). The combination of both drugs significantly decreased the total number of errors to criterion compared to the high and low dose of lazabemide and vehicle (*p* = 0.021, *η* ^2^ = 0.261; *p* = 0.01, *η* ^2^ = 0.313; *p* = 0.004, *η* ^2^ = 0.383). No interactions between lazabemide and group were found (*F*_6,54_ = 0.25, *p* = 0.96) nor did lazabemide itself have any effects on behavioral performance.

By contrast, moclobemide decreased the proportion of perseverative errors (*F*_3,45_ = 3.86, *p* = 0.016 *η* ^2^ = 0.216; Fig. 4c) with the highest dose significantly reducing perseverative errors compared with the vehicle group (*p* = 0.003, *η* ^2^ = 0.428). As shown in Fig. 4d, no main effects or interactions were observed with respect to lazabemide on the proportion of perseverative errors (*F*_3,51_ = 0.72, *p* = 0.55, *F*_6,51_ = 0.37, *p* = 0.90, respectively).

### MAO-A inhibition increases the latency to initiate a new trial following an incorrect response

**Fig. 5:**
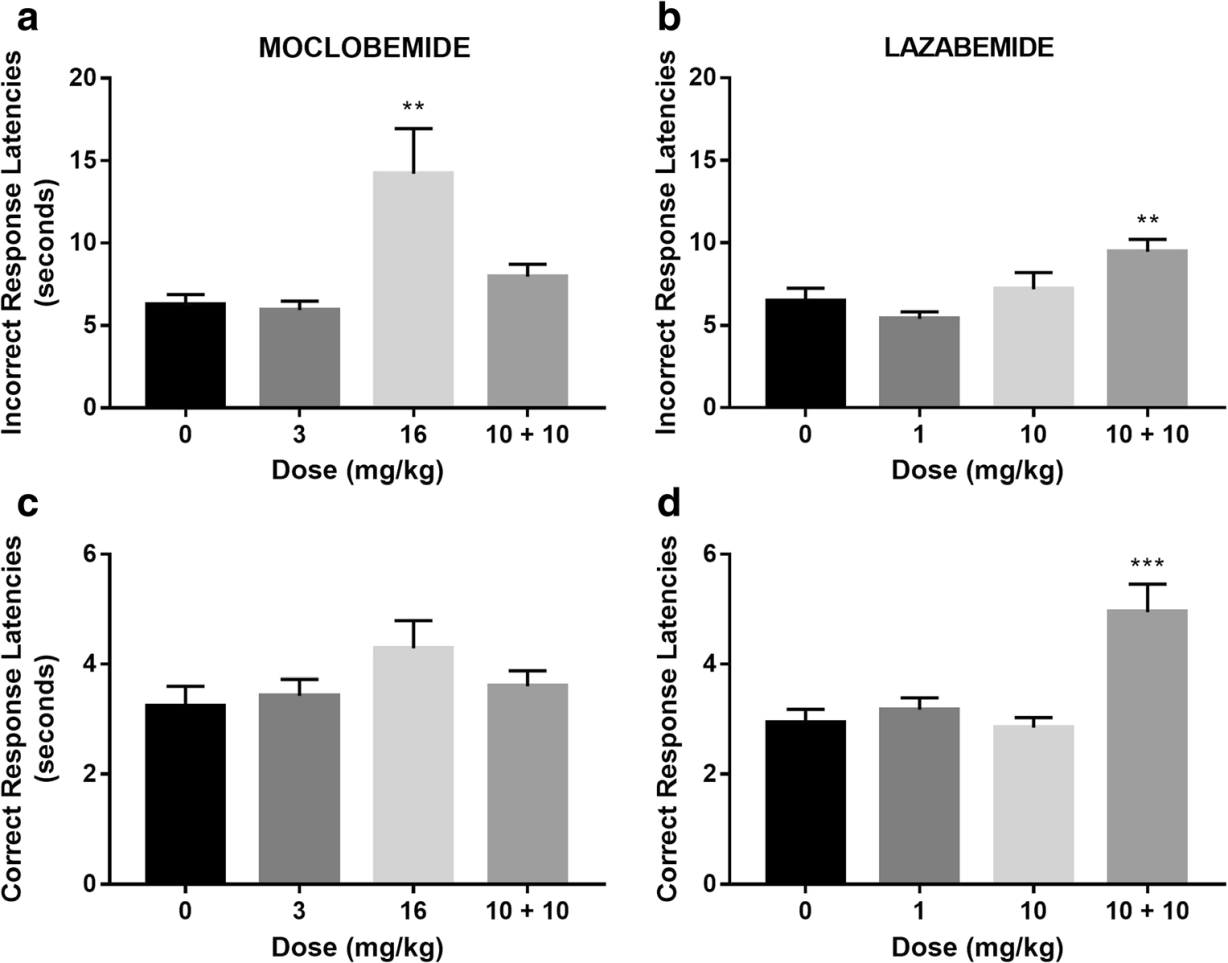
Effects of moclobemide (*n* = 16) and lazabemide (*n* = 20) on response latencies (s) following an incorrect (**a**, **b**) and correct (**c**, **d**) response. Data for two animals was not saved due to a technical failure with the equipment. *p < 0.05, **p < 0.01, ***p < 0.001 versus vehicle

Latencies to initiate a new trial following incorrect and correct responses are shown in Fig. 5. Mixed effect ANOVA with treatment as a within-subject factor and latency type as a between-subject factor revealed a significant difference in the pattern of effects produced by the drug treatment on correct and incorrect responses in the moclobemide group (treatment × latency type interaction *F*_3,87_ = 5.74, *p* = 0.001, *η* ^2^ = 0.165) but not in the lazabemide group (*F*_3,87_ = 1.81, *p* = 0.150). Repeated measures ANOVA revealed a significant main effect of dose for the moclobemide group (*F*_3,45_ = 7.514, *p* = 0.011, *η* ^2^ = 0.334; Fig. 5a) with the highest dose prolonging incorrect response latencies compared with the vehicle group (*p* = 0.004, *η* ^2^ = 0.440), combination treatment (*p* = 0.038 *η* ^2^ = 0.258) and the low dose of moclobemide (*p* = 0.013, *η* ^2^ = 0.345). In addition, incorrect response latencies following the combined drug injections were significantly longer than those following a low dose of moclobemide (*p* = 0.024, *η* ^2^ = 0.297). A similar pattern of results was obtained for the lazabemide group (main effect of dose *F*_3,57_ = 5.622, *p* = 0.002 *η* ^2^ = 0.228; Fig. 5b) with the drug combination again lengthening incorrect response latencies compared with the vehicle group (*p* = 0.012 *η* ^2^ = 0.287).

A different pattern of results was observed with respect to the effects of MAO-A and MAO-B inhibition on correct response latencies. No significant differences were found in the MAO-A group (*F*_3,45_ = 2.164, *p* = 0.105; Fig. 5c), while correct response latencies in the MAO-B group were significantly variable between the different drug groups (*F*_3,57_ = 13.523, *p* = 0.0001, *η* ^2^ = 0.416; Fig. 5d). A combination of both drugs increased the time to initiate a new trial following a correct response compared with the vehicle group (*p* = 0.0001, *η* ^2^ = 0.489).

### MAO-A inhibition facilitates a lose-shift strategy in highly perseverative animals only

A repeated measures ANOVA with treatment as within-subject factor and baseline perseveration (high versus low perseveration) as between-subject factor revealed a significant main effect of drug (*F*_3,24_ = 5507, *p* = 0.005, *η* ^2^ = 0.408) and a significant interactive effect of treatment with the probability of changing a response after a loss trial (*F*_3,24_ = 3373, *p* = 0.035, *η* ^2^ = 0.297). Mean lose-shift probabilities indicated that moclobemide selectively increased the probability of shifting compared with vehicle or combination treatment after a loss trial in highly perseverative but not low-perseverative animals (Table 4). No other significant effects of drug treatment on the win-stay or lose-shift probabilities were observed (all *p* values >0.05).

### MAO-A inhibition strongly increases 5-HT and NE content in OFC, DRN and BLA

**Fig. 6:**
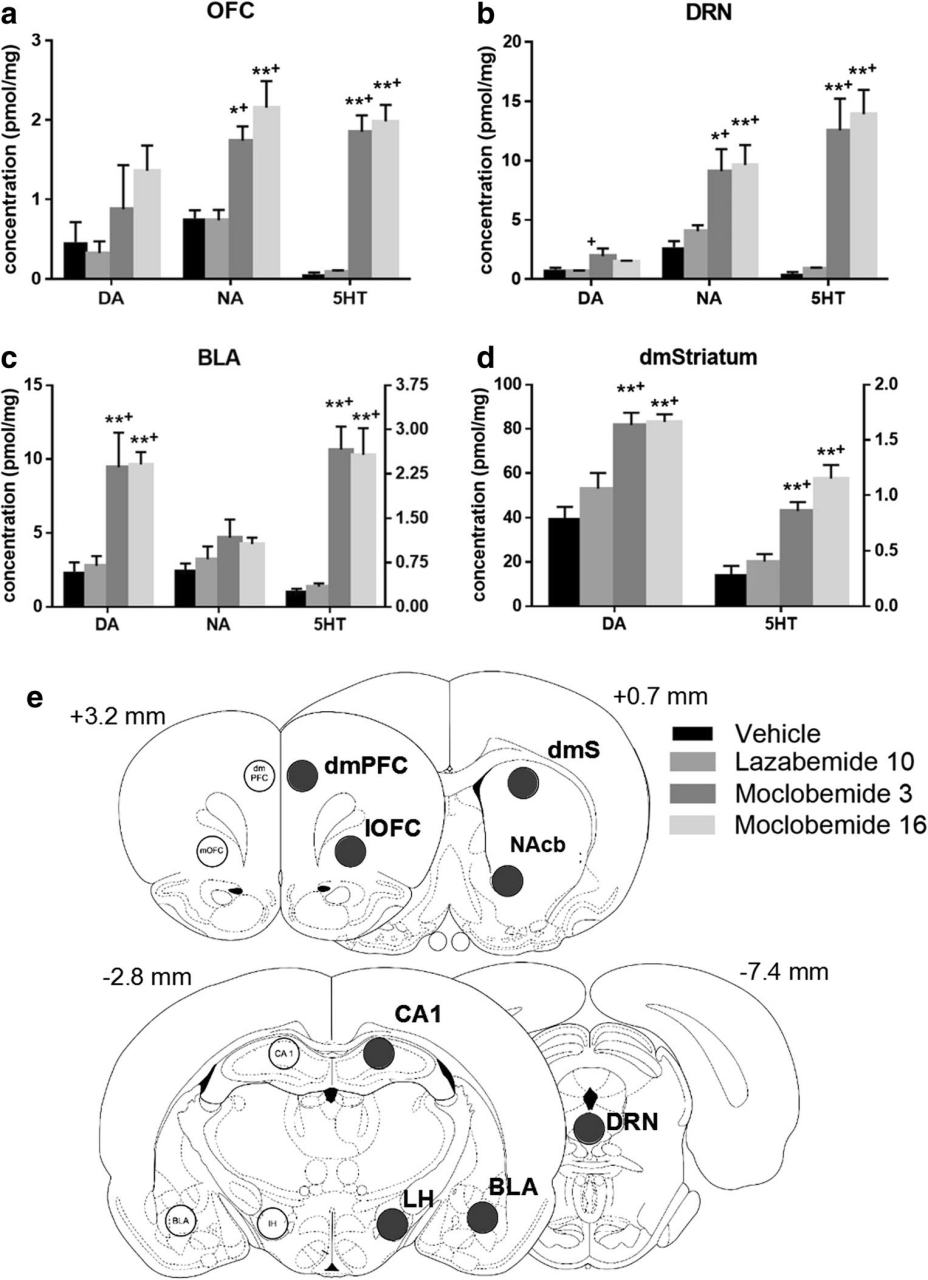
Effects of selective MAO inhibition on monoamine levels in **a** OFC, **b** DRN, **c** BLA, and **d** dorsomedial striatum (pmol/mg tissue). In **c** and **d**, dopamine levels are shown on the left *y*-axis while NA and 5-HT levels are shown on the right *y*-axis. Data are mean values ± SEM. Significance is denoted as follows: *p < 0.05, **p < 0.005 versus vehicle, ^+^ *p* < 0.05 versus lazabemide. **e** Coronal sections showing regions of interest for ex vivo neurochemical analysis of monoamines following vehicle, moclobemide, and lazabemide administration. *dmPFC* dorsomedial PFC, *OFC* orbitofrontal cortex, *dmS* dorsomedial striatum, *NAcb* nucleus accumbens, *BLA* basolateral amygdala, *CA1* hippocampal CA1 region, *LH* lateral hypothalamus, *DRN* dorsal raphé nuclei. Adapted from Paxinos and Watson (2013)

The effects of selective MAO-A and MAO-B inhibition on brain monoamine content are shown in Fig. 6 and Table 3. For each chemical neuromodulator, a separate ANOVA model was tested. A two-way ANOVA with drug treatment and region as between-subject factors revealed significant main effects of treatment (*F*_3,119_ = 82.17, *p* < 0.0001, *η* ^2^ = 0.627) with respect to 5-HT levels. Post hoc analyses of main effects (LSD) show that across all regions of interest, 5-HT levels were significantly higher following both high (16 mg/kg) and low (3 mg/kg) doses of moclobemide than following lazabemide or vehicle injections (all *p* < 0.0001). Notably, lazabemide did not increase 5-HT levels compared with the vehicle group (*p* = 0.6). However, the increase in 5-HT content induced by moclobemide was not uniform across all regions, as revealed by a significant treatment by region interaction (*F*_21,119_ = 15.92, *p* < 0.0001, *η* ^2^ = 0.738). As shown in Fig. 6 and Table 3, average 5-HT levels increased 35 ± 10-fold (±CI_0.95_) following a high dose of moclobemide compared to vehicle in the OFC, 30 ± 13-fold in the DRN, 27 ± 17-fold in the lateral hypothalamus, 9 ± 4-fold in the BLA, and 4 ± 1-fold in the dorsomedial striatum.

**Table 3:** Levels of DA and 5-HT in regions of interest following vehicle (Veh, *n* = 5), lazabemide (L10, *n* = 6), and moclobemide (M3, *n* = 4; M16, *n* = 4) administration

Brain region	Drug	5HT		DA		NA		5HIAA		DOPAC	
BLA	Veh	0.28	(0.24)	2.39	(1.13)	0.63	(0.20)	3.06	(0.39)	4.36	(0.68)
	L10	0.34	(0.22)	2.78	(1.03)	0.81	(0.18)	2.13	(0.35)	4.53	(0.62)
	M3	2.66	(0.27)**^,^***	9.45	(1.26)**^,^***	1.17	(0.22)	1.37	(0.43)*	1.80	(0.76)
	M16	2.57	(0.27)**^,^***	9.64	(1.26)**^,^***	1.07	(0.22)	0.83	(0.43)**^,^***	1.35	(0.76)***
dmPFC	Veh	0.39	(0.17)	0.44	(0.47)	1.25	(0.22)	1.67	(0.39)	0.71	(0.32)
	L10	0.62	(0.16)	0.90	(0.43)	1.74	(0.20)	1.57	(0.35)	0.86	(0.29)
	M3	1.97	(0.19)**^,^***	1.66	(0.53)	2.03	(0.25)	1.15	(0.43)	0.89	(0.36)
	M16	2.20	(0.19)**^,^***	2.25	(0.53)	2.62	(0.25)**	0.77	(0.43)	1.27	(0.36)
dmS	Veh	0.29	(0.08)	39.97	(5.78)					12.92	(1.34)
	L10	0.40	(0.07)	53.03	(5.27)					14.44	(1.23)
	M3	0.86	(0.09)**^,^***	81.71	(6.46)**^,^***					6.55	(1.50)*^,^***
	M16	1.15	(0.09)**^,^***	83.11	(6.46)**^,^***					6.84	(1.50)***
DRN	Veh	0.46	(1.35)	0.80	(0.28)	2.69	(1.08)	6.37	(0.39)	7.85	(3.57)
	L10	0.89	(1.23)	0.66	(0.25)	4.05	(0.99)	6.66	(0.35)	0.96	(3.26)
	M3	12.56	(1.51)**^,^***	1.94	(0.31)***	9.11	(1.21)*^,^***	3.52	(0.43)	0.74	(3.99)
	M16	13.93	(1.51)**^,^***	1.50	(0.31)	9.63	(1.21)**^,^***	2.76	(0.43)***	1.16	(3.99)
Hippo	Veh	1.72	(0.22)	0.21	(0.62)	2.86	(0.39)	1.68	(0.39)	2.03	(0.54)
	L10	0.57	(0.18)	1.22	(0.56)	2.47	(0.36)	1.65	(0.35)	2.14	(0.49)
	M3	1.70	(0.22) *^,^***	0.10	(0.69)	3.54	(0.44)	0.96	(0.43)	0.58	(0.60)
	M16	1.75	(0.22) *^,^***	0.07	(0.69)	3.24	(0.44)	1.01	(0.43)	1.18	(0.60)
lH	Veh	0.19	(0.69)	0.22	(0.14)	3.26	(1.00)	2.26	(0.39)	2.76	(0.95)
	L10	0.29	(0.63)	0.34	(0.13)	4.13	(0.91)	2.43	(0.35)	1.35	(0.86)
	M3	4.97	(0.77)**^,^***	1.72	(0.16)**^,^***	7.77	(1.11)	1.77	(0.43)	1.31	(1.06)
	M16	5.08	(0.77)**^,^***	2.51	(0.16)**^,^***	10.92	(1.11)**^,^***	1.38	(0.43)	0.75	(1.06)
lOFC	Veh	0.06	(0.12)	0.46	(0.24)	0.76	(0.18)	1.91	(0.39)	3.66	(1.11)
	L10	0.09	(0.11)	0.33	(0.22)	0.74	(0.16)	1.32	(0.35)	1.22	(1.01)
	M3	1.85	(0.13)**^,^***	0.88	(0.38)	1.74	(0.20)*^,^***	1.37	(0.43)	3.47	(1.24)
	M16	1.98	(0.13)**^,^***	1.36	(0.27)	2.15	(0.20)**^,^***	1.12	(0.43)	0.78	(1.24)
NAcc	Veh	0.23	(0.09)	24.38	(3.74)			1.23	(0.39)	15.89	(0.99)
	L10	0.26	(0.08)	27.63	(3.42)			1.15	(0.35)	12.82	(0.91)
	M3	0.92	(0.10)**^,^***	47.03	(4.18)**^,^***			0.85	(0.43)	7.10	(1.11)**^,^***
	M16	1.17	(0.10)**^,^***	58.40	(4.18)**^,^***			0.71	(0.43)	6.39	(1.11)**^,^***

Following a significant two-way interaction between region and drug treatment, Bonferroni-corrected post hoc comparisons were carried out to compare treatment effects within each brain region

*p < 0.05; **p < 0.005 versus vehicle (“Veh”); ***p < 0.05 versus lazabemide (“L10”)

MAO inhibition also produced significant changes in DA and NA content (*F*_3,118_ = 33.70, *p* < 0.0001, *η* ^2^ = 0.461; *F*_3,120_ = 29.69, *p* < 0.0001, *η* ^2^ = 0.426, respectively), with moclobemide significantly increasing DA and NA levels compared with both the vehicle and lazabemide groups (all *p* values <0.0001). However, DA and NA increases were not uniform across all areas, as indicated by the significant interaction between region and treatment (*F*_3,90_ = 28.80, *p* < 0.0001, *η* ^2^ = 0.490; *F*_15,90_ = 5.791, *p* < 0.0001, *η* ^2^ = 0.491, respectively). Strongest increases in average DA levels (11-fold) were found in the lateral hypothalamus, with up to 5-fold increases in other regions, including a 2-fold increase in the striatum (Fig. 6; Table 3). Similarly, 3-fold increases in NA were found in the OFC and DRN and up to 2-fold increases in other brain regions. MAO-B inhibition did not significantly affect 5-HT, DA, or NA levels compared to the vehicle treatment as revealed by post hoc contrasts (*p* = 0.60, *p* = 0.06, *p* = 0.24, respectively).

## Discussion

The main findings of this investigation indicate that behavioral inflexibility, as measured by perseverative responding on a spatial reversal learning task, is multidimensional and linked to reduced anxiety and increased levels of circulating plasma 5-HT prior to behavioral training. Inflexible behavior on this task was significantly improved by MAO-A inhibition, but not by MAO**-**B inhibition, and was accompanied by strong increases in 5**-**HT and NE levels in the OFC, DRN, and BLA, as well as longer latencies to initiate a new trial following an incorrect, but not a correct response. These findings collectively indicate that inter-individual variation in behavioral flexibility correlates with low trait anxiety and peripheral measures of serotonergic function and is strongly and selectively modulated by MAO-A inhibition, which putatively may have the effect of strengthening behavioral inhibition in response to recent negative feedback.

Animals exhibiting high levels of perseverative responding during reversals of the instrumental contingency were *less* anxious on the elevated plus maze than low perseveration animals. At first glance, this finding appears to run counter to traditional views that anxiety relief is an important driver for maintaining compulsive behavior in OCD. However, although obsessions and compulsions may be accompanied by anxiety symptoms, and worsened by stress, a prominent causal role of anxiety in OCD for compulsive behavior is controversial and not widely accepted (Fineberg et al. 2010; Hollander et al. 2008; Stein et al. 2010). Moreover, to our knowledge, no studies have hitherto reported the direct relationship between trait-like variation in anxiety and behavioral flexibility in rodents. Nevertheless, consistent with the present study, high trait-like anxiety in marmoset monkeys has been associated with a tendency for improved flexibility on tasks that depend on the anterior OFC and ventrolateral PFC (Shiba et al. 2014). The explanation for the apparent inverse relationship between perseveration and trait anxiety is unclear but may be related to increased vigilance and/or enhanced sensitivity of highly anxious subjects to negative environmental cues and feedback (Bradley et al. 1999; Cisler and Koster 2010). Thus, following a shift in the stimulus-response (S-R) contingency, subjects exhibiting increased anxiety may be less likely to perseverate because their attention is drawn to the previously incorrect (i.e., non-reinforced) stimulus and through increased exploration more readily detect changes in the S-R contingency (Homberg and Lesch 2011). This hypothesis suggests that low-anxious, highly perseverative rats may disregard negative feedback in preference for positive stimuli, and this may be relevant to the beneficial effects of MAO-A inhibition on behavioral flexibility.

A small but significant component of the variance in perseveration was accounted for by plasma levels of 5-HT measured prior to training on the reversal learning task. No associations were found for circulating levels of the 5-HT precursor tryptophan or hormones linked to stress and the hypothalamic-pituitary adrenal axis (NE and corticosterone). While the latter markers provide further separation between perseveration and anxiety and stress responses, our exploratory finding of a positive relationship between perseveration and plasma 5-HT suggests a possible reciprocal relationship between peripheral and central measures of 5-HT function underlying natural variation in behavioral flexibility. Thus, in a recent study, rats stratified for high and low perseverations on an identical spatial reversal task exhibited reduced indices of serotonergic transmission in the DRN and OFC (Barlow et al. 2015). However, the exact mechanisms underlying the apparently opponent relationship between plasma and brain 5-HT remain unclear and would require further studies to directly contrast plasma 5-HT levels with task-related changes in extracellular 5-HT in the brain, in addition to assessing platelet MAO activity (Arrojo et al. 2007).

Using the same paradigm as the present study, we recently reported that high trait-like perseveration in rats is associated both with decreased MAO-A and MAO-B expressions in the dorsal raphé nucleus and increased MAO-A and MAO-B expression in the lateral OFC (Barlow et al. 2015). Highly perseverative animals exhibited reduced 5-HT metabolism and 5-HT_2A_ receptors in the OFC compared with low-perseverative rats (Table 4). In the present study, the selective MAO-A inhibitor moclobemide, but not the MAO-B inhibitor lazabemide, significantly reduced the total number of trials and total errors animals made before they achieved the set criteria for reversal. Although both doses of moclobemide improved general reversal performance, only the highest dose (16 mg/kg) reduced the proportion of perseverative errors. Notably, the higher dose of moclobemide also prominently increased the time rats took to initiate a new trial following an incorrect response but not following a correct response. Similarly, only the high dose of moclobemide increased the probability of high- but not low-perseveration rats to change their response following an incorrect trial. These selective effects on trial outcome tend to rule out mechanisms relating to hyperactivity, a consequence of MAO inhibition (Barbelivien et al. 2001), and may instead indicate increased behavioral resilience to the negative feedback of non-reward or to error monitoring processes often associated in humans with the anterior cingulate cortex (via “error-related negativity”), which is also elevated in patients with OCD (Endrass and Ullsperger 2014).

**Table 4:** Lose-shift probabilities for high (*n* = 5) and low (*n* = 5) perseveration rats that received moclobemide (16 mg/kg, 3 mg/kg), combination of lazabemide and moclobemide, and vehicle

Perseveration	Condition	Pr(shift|loss) ± SEM
High	Moclobemide 16	0.68 ± (0.08)*^,^**
	Moclobemide 3	0.55 ± (0.04)**
	Combination	0.38 ± (0.05)
	Vehicle	0.45 ± (0.06)
Low	Moclobemide 16	0.55 ± (0.08)
	Moclobemide 3	0.68 ± (0.04)
	Combination	0.50 ± (0.05)*
	Vehicle	0.60 ± (0.06)

*p < 0.05 versus vehicle; **p < 0.05 versus drug combination

The neural mechanism underlying the improvement in behavioral indices of cognitive flexibility by moclobemide is unclear but parsimoniously may involve a facilitation in serotonergic transmission in several other brain regions including the OFC and amygdaloid complex (Clarke et al. 2005, 2011; Izquierdo et al. 2016; Rygula et al. 2010). Reversible MAO-A inhibition profoundly increased 5-HT (and NA) content in every region assayed, including the DRN, BLA, and lateral OFC. Changes in DA content were less consistent, however, with significant increases evident only in the BLA and striatum. Central 5-HT plays a critical role in adaptive responses to aversive and threatening stimuli (Cools et al. 2008; Dayan and Huys 2009; Deakin and Graeff 1991) and low levels of 5-HT produced by acute dietary tryptophan depletion lead to negatively-biased decision-making (Cools et al. 2008; Rogers et al. 2003). In rats, 5-HT exerts complex effects on reward sensitivity and negative feedback (Bari et al. 2010; Rygula et al. 2015). For instance, acute 5-HT reuptake inhibition with a high dose of citalopram (10 mg/kg) decreased the sensitivity of rats to negative feedback in a probabilistic reversal learning task and facilitated behavioral flexibility (Bari et al. 2010), while the same dose was found to improve behavioral flexibility on a spatial reversal learning task (Barlow et al. 2015). However, it is less clear how the reported effects of moclobemide on reversal learning relate to measures of anxiety. While MAO-A inhibition produces anxiolytic effects in rats (Caille et al. 1996; Eroğlu and Güven 1998), both acute activation of the serotonergic dorsal raphé nucleus (Urban et al. 2015) and acutely administered selective 5-HT inhibitors (Birkett et al. 2011; Mombereau et al. 2010) increase anxiety. These findings thus suggest that the anxiolytic effects of MAO-A inhibitors, including moclobemide, are unlikely to be due to acute increases in 5-HT transmission and implicate as a result other neurotransmitter systems in this effect, including NA (see Eroğlu and Güven 1998). Since low anxiety was associated with high trait-like levels of perseveration, it is unlikely that moclobemide, with presumed anxiolytic effects, improved reversal learning by reducing anxiety.

Although citalopram and moclobemide both facilitated reversal performance, the effect size of moclobemide was significantly larger than that of citalopram (compare Barlow et al. 2015). This difference may be explained by the effects of SSRIs to simultaneously exert biphasic inhibitory and facilitating effects on 5-HT transmission through blockade of 5-HT reuptake and activation of inhibitory somatodendritic 5-HT_1A_ autoreceptors in the DRN (Sprouse and Aghajanian 1987). Thus, high doses of citalopram have the effect of increasing extracellular levels of 5-HT in the PFC, as measured by in vivo microdialysis (Invernizzi et al. 1992) but not at lower doses, which activate inhibitory 5-HT autoreceptors and diminish the activity of serotonergic neurons in the DRN (Gardier et al. 1996). In contrast, MAO-A inhibition does not affect the function of 5-HT autoreceptors, even after long-term administration, (Blier et al. 1988), and consistently increases 5-HT levels by inhibiting its decomposition (Kumagae et al. 1991; Stahl 2015). Thus, unlike citalopram that dose-dependently impairs and improves reversal learning (Bari et al. 2010), moclobemide apparently exerts monophasic effects on 5-HT transmission and strongly promotes behavioral flexibility.

In addition to its effects on 5-HT, MAO-A inhibition also increased NE levels in the lateral OFC, PFC, and DRN. While some studies report that acute NE reuptake inhibition and α-2A receptor activation improves intradimensional (ID) reversal performance (Seu and Jentsch 2009; Steere and Arnsten 1997), other studies using similar manipulations report effects on extradimensional reversal learning (e.g., Bradshaw et al. 2016). Moreover, central NE depletion did not impair performance on a taste/tactile reversal task (Jarbe et al. 1988) and NE efflux increased only weakly in the rat medial PFC during the reversal phase of a serial reversal learning task (Van Der Meulen et al. 2007), suggesting that NE may be less important for behavioral adaptation following changing stimulus-reward contingencies than for general attentional processes needed for successful set-shifting (Cain et al. 2011; Tait et al. 2007; Totah et al. 2015).

Contrasting with the effects of moclobemide, selective MAO-B inhibition with lazabemide produced no significant effects on task performance nor did this compound affect tissue levels and turnover of 5-HT, DA, and NE in a number of cortical and subcortical regions. These negative findings were very unlikely to be due an insufficient dose of lazabemide since much lower doses were reported to inhibit ex vivo MAO-B activity by over 80% while leaving MAO-A activity unaffected (2 mg/kg (Henriot et al. 1994); 1 mg/kg (Jolkkonen et al. 2000)). Moreover, the selected high dose of lazabemide (10 mg/kg) has been shown to produce robust behavioral effects in other settings (Maki et al. 2000). Rather, the absence of significant effects of lazabemide in the present study is more consistent with its high selectivity for the MAO-B subtype that preferentially targets trace amines (Shih and Thompson 1999). The singular contribution of MAO-A inhibition to promoting behavioral flexibility was confirmed by the combination treatment of moclobemide and lazabemide, which mimicked the effects of moclobemide alone.

In conclusion, our findings demonstrate that selective and reversible inhibition of MAO-A but not MAO-B activity enhances behavioral indices of cognitive flexibility regardless of baseline flexibility on a spatial-discrimination reversal learning task. Our results show, apparently for the first time, that natural variation in behavioral flexibility is partly predicted by reduced measures of trait-like anxiety and increased plasma levels of 5-HT. Since cognitive flexibility is impaired in OCD (Watkins et al. 2005) and unaffected first-degree relatives of OCD patients (Chamberlain et al. 2007), the index of perseveration used in the present study may represent an endophenotype to support a deeper understanding of etiological mechanisms in OCD and related disorders. Our findings specifically implicate MAO-A in modulating cognitive flexibility and encourage further investigations of this ubiquitous enzyme as a target for diagnosis and treatment.
